# Predicting the Occurrence of Cave-Inhabiting Fauna Based on Features of the Earth Surface Environment

**DOI:** 10.1371/journal.pone.0160408

**Published:** 2016-08-17

**Authors:** Mary C. Christman, Daniel H. Doctor, Matthew L. Niemiller, David J. Weary, John A. Young, Kirk S. Zigler, David C. Culver

**Affiliations:** 1 Departments of Biology and of Statistics, University of Florida, Gainesville, Florida, and MCC Statistical Consulting LLC, Gainesville, Florida, United States of America; 2 U. S. Geological Survey, Reston, Virginia, United States of America; 3 Illinois Natural History Survey, Prairie Research Institute, University of Illinois at Urbana-Champaign, Champaign, Illinois, United States of America; 4 U. S. Geological Survey, Leetown Science Center, Kearneysville, West Virginia, United States of America; 5 Department of Biology, The University of the South, Sewanee, Tennessee, United States of America; 6 Department of Environmental Science, American University, Washington, District of Columbia, United States of America; University of New England, AUSTRALIA

## Abstract

One of the most challenging fauna to study *in situ* is the obligate cave fauna because of the difficulty of sampling. Cave-limited species display patchy and restricted distributions, but it is often unclear whether the observed distribution is a sampling artifact or a true restriction in range. Further, the drivers of the distribution could be local environmental conditions, such as cave humidity, or they could be associated with surface features that are surrogates for cave conditions. If surface features can be used to predict the distribution of important cave taxa, then conservation management is more easily obtained. We examined the hypothesis that the presence of major faunal groups of cave obligate species could be predicted based on features of the earth surface. Georeferenced records of cave obligate amphipods, crayfish, fish, isopods, beetles, millipedes, pseudoscorpions, spiders, and springtails within the area of Appalachian Landscape Conservation Cooperative in the eastern United States (Illinois to Virginia and New York to Alabama) were assigned to 20 x 20 km grid cells. Habitat suitability for these faunal groups was modeled using logistic regression with twenty predictor variables within each grid cell, such as percent karst, soil features, temperature, precipitation, and elevation. Models successfully predicted the presence of a group greater than 65% of the time (mean = 88%) for the presence of single grid cell endemics, and for all faunal groups except pseudoscorpions. The most common predictor variables were latitude, percent karst, and the standard deviation of the Topographic Position Index (TPI), a measure of landscape rugosity within each grid cell. The overall success of these models points to a number of important connections between the surface and cave environments, and some of these, especially soil features and topographic variability, suggest new research directions. These models should prove to be useful tools in predicting the presence of species in understudied areas.

## Introduction

Species distribution models (SDMs) have become a fundamental tool used to derive geographic ranges of species and to quantify relationships between species and their environment from occurrence records (usually either presence or presence/absence) and environmental datasets, often bioclimatic variables [1,2]. SDMs have been applied to a wide range of terrestrial and aquatic taxa, and their outcomes are commonly used to inform decisions for a range of applications in ecology, biogeography and conservation (reviewed in [3]), such as management of threatened and endangered species, predicting impacts of future climatic change, and predicting biological invasions. However, a methodological constraint of SDMs is insufficient occurrence data over a species’ distribution (i.e., niche space), as distributional data are often sparse or unevenly distributed across a species’ range. Such limited distribution data may lead to spurious predictions, particularly at continental or global scales [4,5]. Regrettably, many species that are at risk of extinction and are targets of conservation have ranges too restricted for large-scale correlative SDMs, severely limiting their use in defining conservation priorities. For example, Platts et al. [5] reported that 55 percent of 733 amphibian species in sub-Saharan Africa had too few occurrence records for correlative SDMs, including 92 percent of species at elevated risk of extinction. To overcome this rare species problem, several new approaches have been developed, such as hierarchical approaches that combine species-specific and community models [6–8].

Caves contain a unique and phylogenetically diverse fauna. Successful long-term survival and reproduction in caves is contingent upon a strong environmental filter through which surface-dwelling populations must pass. One important element of this filter is the complete absence of light, and the surface ancestors of troglobionts (obligate aquatic and terrestrial cave-dwelling taxa) are usually species that themselves do not have a strong dependence on light, such as species living in forest leaf litter [9]. However, knowledge of the environmental filter and of the biology of potential colonizing species is not sufficient to predict the composition of the cave fauna, even at higher taxonomic levels (e.g., order or family). The fauna of caves is reduced in taxonomic richness compared to surface communities, especially at higher taxonomic levels [10].

Furthermore, the taxonomic composition of cave communities varies geographically. At the species level, differences in taxonomic composition are striking. In the eastern U.S., most troglobionts have highly restricted distributions, with many known from a single cave [11,12], and none have been subjects of SDMs. In Europe, α-diversity (local diversity) is only a minor component of regional aquatic subterranean diversity [13]. In the highly biodiverse Dinaric karst of central Europe, two beetle families, both with more than 80 species in the region, have different centers of richness and different overall geographic ranges [14].

Historical factors combined with highly restricted opportunities for dispersal likely play a large role in explaining the distribution of subterranean species and genera. But, for higher taxonomic categories, that typically represent a large number of subterranean lineages [15], other factors may come into play, such as habitat availability (e.g., number of caves or amount of karst), geographic position (e.g., distance from the maximum extent of Pleistocene glaciation), climatic variables (e.g., mean annual temperature and precipitation), and regional hydrology (e.g., base flow index [BFI], which is the ratio of annual base flow in a river to the total annual runoff).

Most previous studies in caves have focused on species richness [16], (e.g., the effect of geological barriers on distribution in the Tennessee cave fauna [12], and range sizes of groundwater (aquatic cave and interstitial) species in Europe [17]). In the European study, range sizes of species increased with latitude above 43°N, and that temperature change since the Last Glacial Maximum accounted for substantially greater variation than precipitation or habitat availability and variability.

In this study, we address the fundamental ecological question of whether surface environmental variables can accurately predict the presence and absence of troglobiotic species. Because occurrence data are sparse for most troglobiotic species, we take a community-level modeling strategy that combines data from multiple related species of the same functional classification in cave ecosystems with environmental predictors. We chose nine faunal groups (five terrestrial and four aquatic) that are common components of terrestrial and aquatic cave communities: ground beetles (Carabidae), millipedes, pseudoscorpions, spiders, and springtails for terrestrial species groups, and amphipods (Crangonyctidae and Gammaridae), isopods (Asellidae), crayfishes (Cambaridae), and fishes (Amblyopsidae) for aquatic species groups.

The ways that the above-ground environment influence the cave environment are multifaceted, and include short-term (ecological) and long-term (evolutionary) effects. Evolutionary effects primarily influence the likelihood of successful colonization of caves, while ecological effects primarily influence the probability of persistence of populations. Both of these effects are at least partially dependent upon the age of the karst and the caves themselves.

Geological or paleontological evidence that may provide age constraints on karst evolution in the Appalachian regions are sparse, but some data exist. For example, the Pipe Creek sinkhole fauna provides an age constraint on the Interior Lowland Plateaus in Indiana, dating back to the Early Pliocene [18,19]. The Gray Fossil Site in eastern Tennessee places an age constraint on the karst of the Appalachian Mountains to be at least as old as the Eocene based on palynological work [20], and palynology of the lignite deposit at Pond Bank, Pennsylvania, possibly extends this age to be as old as the upper Cretaceous [21]. Species limited to caves in karst terrain are unlikely to be older than the karst landscape in which they reside. Phylogenetic analyses coupled with divergence time estimates suggest that some species/species groups are relatively young, such as *Gyrinophilus* cave salamanders (Pleistocene, [22,23]), amblyopsid cavefishes (*Typhlichthys* and *Amblyopsis*, late Pliocene to Pleistocene, [24,25]), while other groups appear quite old such as *Bactrurus* amphipods (30–58 mya, [26,27]. A similar broad range of ages has been reported for aselloid isopods in Europe [28].

We examined a range of such influences, including direct measures of available habitat (amount of karst-bearing rocks), topography, geographic position, climate, hydrology, and soil characteristics. In addition to the intrinsic ecological interest in understanding the links between the subterranean fauna and surface environmental factors, there is a more practical interest for conservation practitioners. The troglobiotic fauna is one of the most highly endemic faunas known. The problem is that a relatively small percentage of caves have been bio-inventoried, even in very well studied areas like Slovenia, where less than 20 percent of the 7,000 known caves have been inventoried [29]. In the eastern U.S., the percentage is even lower. If knowledge of surface conditions can help predict the presence of subterranean fauna, it will be an extremely useful tool in conservation planning.

## Materials and Methods

### Study Area

**Fig 1:**
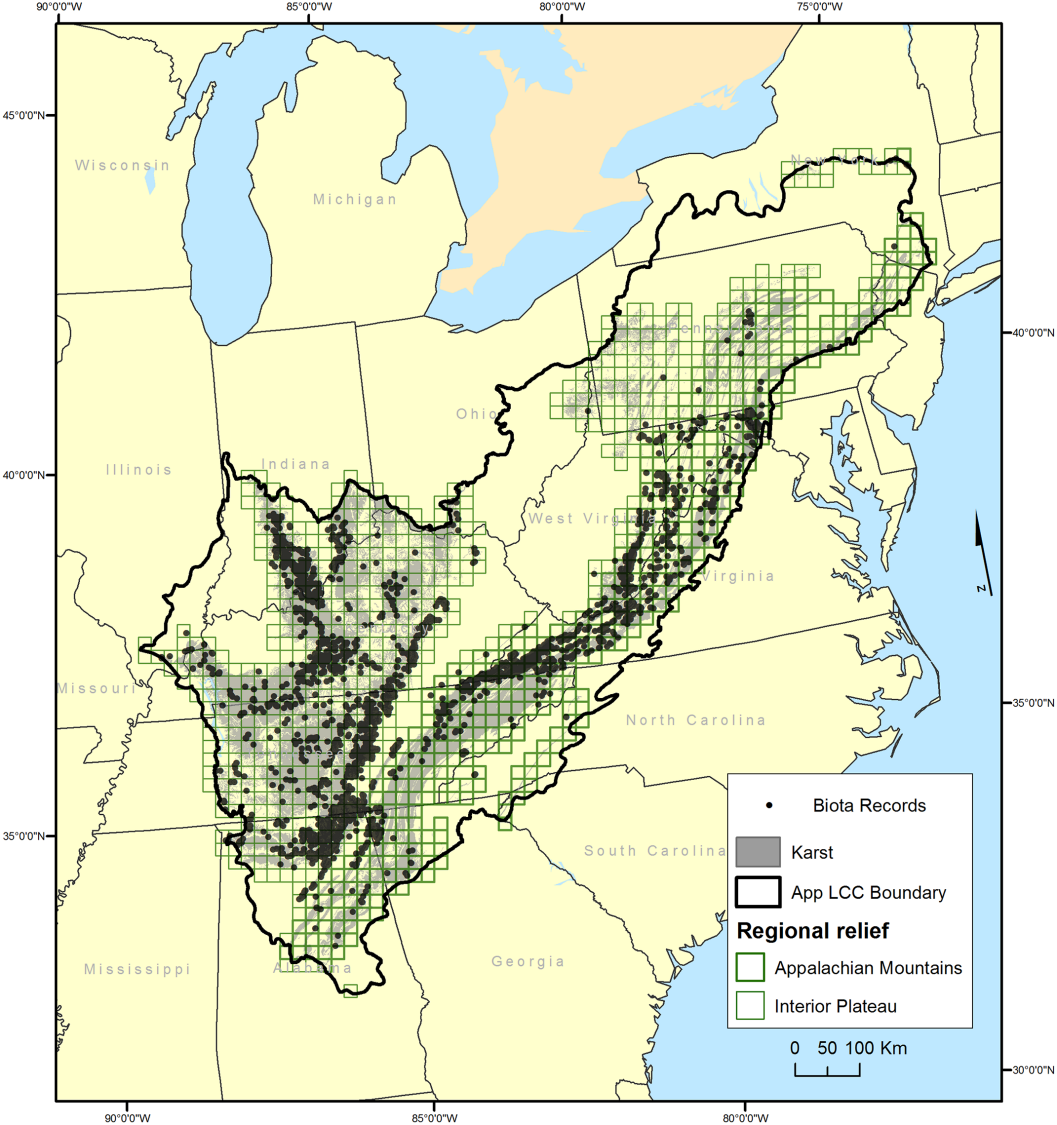
Map of study area. Areas of karst are shaded and overlain by a 20 x 20 km grid, with karst grid cells in the Appalachian Mountains and in the Interior Low Plateau outlined in green. Black dots show caves with known troglobionts. Adapted from [31].

The Appalachian Landscape Conservation Cooperative (LCC) is one of 22 private-public partnerships established by the United States Fish and Wildlife Service to aid in developing landscape scale solutions to conservation problems (http://www.lccnetwork.org). The Appalachian LCC comprises part or all of 15 States, and includes extensive areas of karst within the Interior Low Plateau, Southwestern-Central and North Central Appalachians, and Ridge and Valley Level III ecoregions. For this study, we have combined the latter three ecoregions into a unit we are calling the Appalachian Mountains because they have similar physiography (Fig 1). The Appalachian LCC includes all major karst regions east of the Mississippi River except for the Florida Lime Sinks, and more than half the caves known in the United States [30]. Karst areas within the Appalachian LCC were delineated using the data compiled by the U.S. Geological Survey of karst in the United States [31]. The ecoregions of the Interior Low Plateau and Appalachian Mountains were analyzed separately because of the well documented faunal [12,32] and geological differences, including regional lithology and cave geometry [33].

### Obligate Cave-Dwelling Fauna Occurrences

We gathered occurrence data on the cave-limited terrestrial and aquatic troglobiotic species from a variety of sources, including the scientific literature, existing biodiversity databases, personal records of the authors, and museum accessions. Literature records were compiled from peer-reviewed journals, books, conference proceedings, theses and dissertations, government reports, and caving organizational newsletters. This included keyword searches of ISI Web of Science, Zoological Record, and Google Scholar. We also acquired occurrence records from biodiversity databases maintained by State Natural Heritage programs and The Nature Conservancy (for Tennessee). All occurrence records were incorporated into an ArcGIS (v10) shapefile. We attempted to georeference each occurrence record from published sources and with the assistance of state cave surveys. We included georeferenced records for troglobiotic taxa for which species identifications were not available but were identified at least to the level of genus. The resulting database had 11,134 records at the time of the analysis (July 2015), 10,518 of which had geographic coordinates available for mapping, and including 9833 records identified to species for 750 taxa. These are not public records due to the sensitivity of the information, data sharing agreements, as well as restrictions under the Federal Cave Resources Protection Act of 1988. Researchers interested in this data set may contact ML Niemiller for data access. Occurrence records were translated into a presence-absence matrix, and identified by location (e.g., grid cell id) within a 20 x 20 km grid generated using GIS and encompassing the study area. Abundance records were generally not available, and when the number of individuals in a collection was known, it was unclear whether this reflected abundance, or, more commonly, a limit on the number of individuals collected due to conservation concerns. Data for each of the nine species groups were pooled before modeling (i.e., ‘classification-then-modeling’ approach [34]). Endemism was defined in relationship to the 20 x 20 km grid cells. A cell with at least one species endemic to that grid was counted as a grid cell occupied by endemic taxa.

### Predictor Variables

The following variables were calculated for each 20 x 20 km grid cell and used as predictors of occurrence of the cave-dwelling fauna:

1. Percent area of carbonate bedrock representing potential karst, calculated from the USGS national karst map database [31].
2. Length (and log length) of the edge between carbonate and non-carbonate bedrock, calculated from the USGS national karst map database [31]. For the purposes of analysis, the length and log length of the karst/non-karst boundary are treated as one variable.
3. Mean and standard deviation of elevation, using a 1 x 1 km grid, interpolated from 90 m resolution Shuttle Radar Topography Mission (SRTM) elevation data, available at https://lta.cr.usgs.gov/SRTM2.
4. Mean and standard deviation of Topographic Position Index (TPI), a measure of local relief which represents the difference between the mean elevation of a local 1 x 1 km grid cell and the mean elevation of cells in a surrounding neighborhood. It was calculated using a moving window of an annulus with a 2 km inner radius and a 10 km outer radius, using the Land Facet Corridor Designer Extension for ArcGIS [35].
5. Geographic position, using the centroids of the 20 x 20 km cells.
6. Mean and standard deviation of surface air temperature, calculated from 30-year climate normals (1960–1990) at 1 km resolution and available from the WorldClim bioclimatic dataset (http://www.worldclim.org/bioclim).
7. Mean and standard deviation of precipitation, calculated from 30-year climate normals (1960–1990) at 1 km resolution and available from the WorldClim bioclimatic data set (Available: http://www.worldclim.org/bioclim).
8. Mean and standard deviation of base flow index (BFI), the component of stream flow that can be attributed to groundwater discharge into streams, and expressed as a percentage. The raw data are from the USGS Open File Report. Available: http://water.usgs.gov/GIS/metadata/usgswrd/XML/bfi48grdl.xml. Values for 20 x 20 cells were interpolated from BFI point values estimated from USGS stream gages [36] and computed using an automated hydrograph separation program (Available: http://www.usbr.gov/pmts/hydraulics_lab/twahl/bfi/index.html).
9. Above ground forest biomass in megagrams (Mg) per hectare, from data, Available: http://data.fs.usda.gov/geodata/rastergateway/biomass/index.php.
10. Soils data originally derived from the USDA NRCS STATSGO soils database, Available: http://water.usgs.gov/GIS/metadata/usgswrd/XML/ussoils.xml, including attributes of soil water capacity, permeability, depth to water, thickness, bulk density, and percent organic matter. A principal components analysis (PCA) was performed with the software SAS v9.4 (SAS Institute Inc.) using these six variables, and resulted in three PC axes that were used in further analysis (Table 1). The first axis reflects water retention and transmission capacity of the soil; the second reflects the organic content of the soil (which correlates with shallow water tables); and the third reflects the parent material which influences thickness and density. The three axes explained 74 percent of the total variation in the data.

**Table 1:** Factor loadings of soil characteristics in the 20 X 20 km grid for the study of the first three principle components.

	Component 1	Component 2	Component 3
Water capacity	0.5168	0.1906	-0.4101
Permeability	-0.4989	-0.1926	0.4884
Depth to water	-0.4241	0.5120	-0.1725
Thickness	0.4416	0.0059	0.5243
Bulk density	0.3071	0.3566	0.5290
% organic matter	0.1222	-0.7330	-0.0938

### Statistical Analysis

All statistical analyses were done using PROC LOGISTIC in SAS v9.4 (SAS Institute Inc.). We converted the counts of records in each 20 x 20 km grid cell for each species group to an indicator variable of presence or absence of the species group in each cell. Analyses were limited to those cells with at least one record of a troglobiont, irrespective of group. This eliminated non-karst areas, which have no karstic caves, as well as karst areas either without known caves or without biological records. Zeroes in the analysis were thus cells with troglobionts, but none from the group being considered. It is unlikely that there is bias of these zeroes with respect to the physical variables used in the analysis. There may be a collection bias in that some States are better sampled (e.g., Maryland and Tennessee) than others (e.g., Pennsylvania and New York) but this does not result in the “true zeroes” as defined above.

A selection procedure based on all possible combinations of predictors in logistic regression models was used to identify a suite of predictive variables. For each possible set of predictors (2^20^ combinations), a likelihood score (chi-square) statistic was calculated. The scores for the models were plotted against the number of predictors. The best model was the model with the highest likelihood score before the inflection point, where adding another variable had little effect on increasing the likelihood score. This model was then reviewed for adequacy using receiver operating characteristic (ROC) curves, Hosmer-Lemeshow lack of fit test [37], and the p-values of the Wald tests for significance of the predictor variables. Finally, a leave-one-out cross-classification table was used to identify the optimal cutoff for converting predicted probabilities to predicted presence or absence. Optimal is defined here as the cutoff value with the highest joint sensitivity and specificity. The cutoff was used to convert the predicted probabilities to predicted occurrences; these were used to develop confusion matrices indicating the proportion of observations misclassified by the model.

For each species group, the best model was used to predict the probability that the group would be observed in all grid cells for which karst is present. The coefficients of the models are reported as standardized estimates for comparative purposes.

## Results

**Fig 2:**
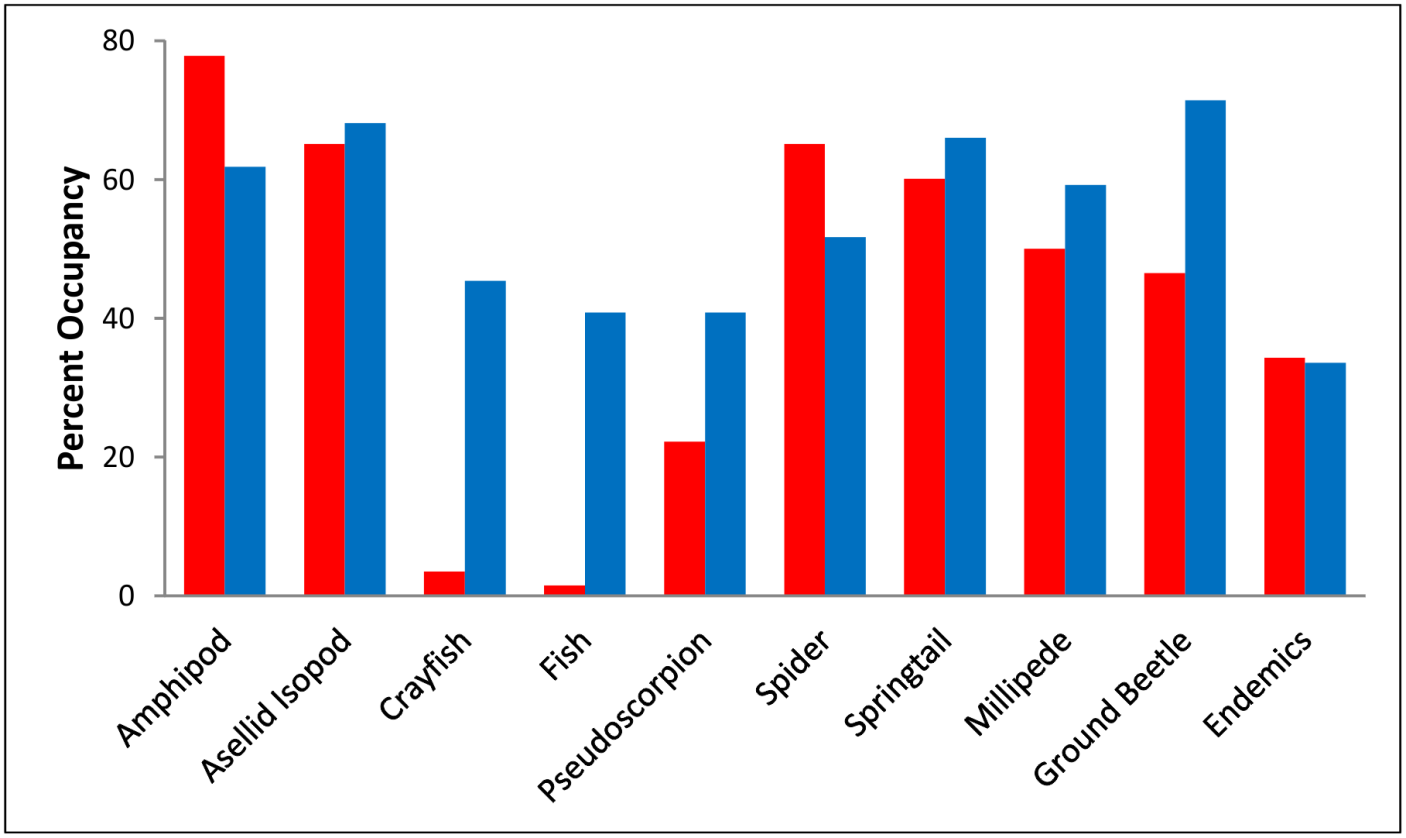
Frequency of occupancy of different groups in cells with at least one troglobiotic species. Values for the Interior Low Plateau are shown in blue, and values for the Appalachian Mountains are shown in red.

A total of 436 20 x 20 km cells had at least one troglobiotic species reported from caves (Fig 1). The highest frequency of cell occupancy was 77.8 percent by amphipods in the Appalachian Mountains and 71.4 percent by ground beetles in the Interior Low Plateau. Fishes were nearly absent from the Appalachian Mountains; they occur only in three grid cells, all near the boundary with the Interior Low Plateau. Crayfishes were nearly as scarce in the Appalachians, occurring in only seven grid cells, all occupied by a single species from West Virginia. Endemic taxa were found in 33.5 percent of grid cells, a remarkably high frequency (Fig 2).

Significant explanatory variables for each of the nine species groups (amphipods, isopods, crayfish, fish, ground beetles, millipedes, pseudoscorpions, spiders, and springtails) and single cell endemics are summarized in Table 2.

**Table 2:** Explanatory variables in the final model for each combination of taxonomic group and region (ILP = Interior Low Plateau, App = Appalachians Mounntains).

Taxon	NS Region	Mean Precipitation	SD Precipitation	Mean Temperature	SD Temperature	Mean Elevation	SD Elevation	Mean Mg/ha	SD Mg/ha	Mean BFI	SD BFI	Mean TPI	SD TPI	% Karst	Edge Length	log(Edge Length)	X Coordinate	Y Coordinate	Soil1	Soil2	Soil3
**Amphipod**	**App**	0.36*		-0.89*		-0.64*						0.29*	0.50*	0.56*							
	**ILP**										-0.17*	0.16*	0.58*	0.37*			-0.22*	0.53*			
**Asellid Isopod**	**App**	0.58*								0.25*								0.94*	-0.56*	0.92*	-0.34*
	**ILP**			1.69*		0.95*		0.39*					0.31*	0.44*			-0.53*	1.65*	0.22*		
**Crayfish**	**App**			-1.15*						-1.98*							4.54*	-5.36*			-1.00*
	**ILP**		-0.84*			0.69*				0.52*	-0.40*		1.04*	0.55*			-0.80*	0.37*			-0.27*
**Fish**	**ILP**	0.88*		-2.03*		-0.85*		0.34*			-0.19*			0.66*	-0.34*			-1.75*			
**Pseudoscorpion**	**App**								0.37*	-0.64*						0.32*	1.20*	-1.29*		0.46*	
	**ILP**	0.33*	-0.58*								-0.23*		0.99*	0.28*		0.17*					
**Spider**	**App**					0.36*	-0.52*			-0.25*		-0.23*	0.77*							0.29*	
	**ILP**							0.35*	0.42*		-0.15			0.22*	-0.28*			0.26*			
**Springtail**	**App**		0.42*			0.22*	-0.85*					-0,33*	0.98*							0.29*	
	**ILP**				-0.39			0.09	0.16*				0.49	0.15				-0.15*		0.14	-0.15
**Millipede**	**App**					0.77*		-0.69*	-0.23*	-0.59*			0.66*	0.29*				-0.49*			
	**ILP**		-0.60*	-0.54			0.55*	0.42*			-0.24*			0.45*				-0.82*			
**Ground Beetle**	**App**					0.51*		-0.57*		-0.59*			0.48*								-0.33*
	**ILP**	0.54*		1.50*		0.57*												1.80*		0.56*	-0.34*
**Endemics**	**App**			-1.40*	-0.55*	-0.90*		0.28				-0.17	1.07*				-1.07*			0.41*	-0.17
	**ILP**	-0.62*	-0.61*	0.82*		0.33			0.19*				0.78*	-0,15							

Values listed are the standardized estimates of the coefficients, and indicate the relative strength of the relationship. An asterisk indicates that the effect is significant (p < .10). BFI is base flow index, Mg/ha is above ground carbon in megagrams per hectare, TPI is topographic position index. See text for details.

Four of the 19 variables had a consistent negative effect in those cases where there was any significant effect—the standard deviation of the base flow index (BFI), PC1, and PC3 for soil. Standard deviation of BFI measures the spatial variability of baseflow contributions to streams, which reflects the diversity of hydrogeologic environments within a grid cell. This would indicate that regions of more variable hydrogeologic conditions will result in a lower probability of species occurrence. PC1 is most heavily loaded with soil water capacity and permeability (negatively, Table 1), and overall is a measure of soil drainage. This negative relationship between group occurrence and soil drainage only occurred once (for the asellid isopods); the other 15 cases were not significant (Table 2). PC3 is most heavily loaded with soil thickness and density, suggesting that sites with thin soils are more likely to have cave animals.

There were environmental variables with a consistent positive relationship with the presence of the different groups of organisms—mean precipitation, standard deviation of TPI, percent karst, and PC2 of soil. Mean precipitation may be related to productivity, especially net primary productivity, and in turn allochthonous organic matter inputs, which may be limiting for many species [16]. Likewise, amount of available habitat (measured by the percent of the areal extent of karst), may be limiting, at least in terms of species richness [38]. PC2 of soil is positively loaded on depth to water and negatively on organic matter, thus it is a parameter that measures the significance of well-drained soils with relatively lower organic matter. The TPI itself is a measure of the relative local elevation position of each square kilometer (a local high or low with respect to the surrounding terrain), thus its standard deviation within a 20 x 20 km cell is a measure of landscape roughness, or rugosity. Latitudinal position was among the variables that showed an inconsistent effect with respect to positive or negative impact on group occurrence.

There were also differences between the Interior Low Plateau and the Appalachian Mountains with respect to variables affecting presence or absence of particular species groups (Table 3). In the Interior Low Plateau, percent karst, y-coordinate (latitude), and the standard deviation of BFI were the most important, while in the Appalachian Mountains mean BFI, mean elevation, and standard deviation of TPI were most likely to affect the likelihood of the presence of a particular species group. For aquatic species groups (amphipods, asellid isopods, crayfishes, and fishes), latitude and percent karst were most important, while the standard deviation of TPI was most important for terrestrial species.

**Table 3:** Cross-classification results from each model. Prob level = value of the cutoff of the probability of observing presence used to classify each prediction into either an event (presence) or non-event (absence). Correct = number of events and non-events properly classified when comparing predicted to observed. Incorrect = number of non-events improperly classified as events and vice versa. Percentages Correct: overall percentage of observations correctly classified. Sensitivity: percentage of actual events correctly identified as such and is complementary to the false negative rate. Specificity: percentage of non-events which are correctly identified as such and is complementary to the false positive rate. False Positive Percentage: percent of predicted event responses that were observed as nonevents. False Negative Percent: percent of predicted nonevent responses that were observed as events.

Taxon	Region	Prob Level	Correct		Incorrect		Percentages				
			Event	Non-Event	Event	Non-Event	Correct	Sensitivity	Specificity	False Positive	False Negative
**Amphipod**	**Mountains**	**0.22**	153	3	41	1	78.8	99.4	6.8	21.1	25
	**Plateaus**	**0.38**	132	32	59	15	68.9	89.8	35.2	30.9	31.9
**Asellid Isopod**	**Mountains**	**0.34**	122	11	58	7	67.2	94.6	15.9	32.2	38.9
	**Plateaus**	**0.32**	158	18	58	4	73.9	97.5	23.7	26.9	18.2
**Crayfish**	**Mountains**	**0.04**	6	168	23	1	87.9	85.7	88	79.3	0.6
	**Plateaus**	**0.52**	71	104	26	37	73.5	65.7	80	26.8	26.2
**Fish**	**Plateaus**	**0.44**	71	103	38	26	73.1	73.2	73	34.9	20.2
**Pseudo scorpion**	**Mountains**	**0.44**	15	140	13	29	78.7	34.1	91.5	46.4	17.2
	**Plateaus**	**0.62**	39	131	8	58	72	40.2	94.2	17	30.7
**Spider**	**Mountains**	**0.34**	120	13	56	9	67.2	93	18.8	31.8	40.9
	**Plateaus**	**0.36**	108	49	66	15	66	87.8	42.6	37.9	23.4
**Springtail**	**Mountains**	**0.4**	106	36	43	13	71.7	89.1	45.6	28.9	26.5
	**Plateaus**	**0.36**	156	5	76	1	67.6	99.4	6.2	32.8	16.7
**Millipede**	**Mountains**	**0.48**	74	63	36	25	69.2	74.7	63.6	32.7	28.4
	**Plateaus**	**0.38**	132	33	64	9	69.3	93.6	34	32.7	21.4
**Ground Beetle**	**Mountains**	**0.46**	65	70	36	27	68.2	70.7	66	35.6	27.8
	**Plateaus**	**0.28**	169	8	60	1	74.4	99.4	11.8	26.2	11.1
**Endemics**	**Mountains**	**0.6**	20	121	9	48	71.2	29.4	93.1	31	28.4
	**Plateaus**	**0.52**	25	145	13	55	71.4	31.3	91.8	34.2	27.5

For endemism, the explanatory variables included 13 of the 19 predictor variables; only the standard deviation of elevation, mean and standard deviation of BFI, length of the karst/non-karst edge, and PC1 for soil were not informative given the remaining variables in the model. However, the model performed poorly at predicting the occurrence of endemics (Table 3).

In general, the predictive models were able to predict presence or absence correctly between 66 and 87.9 percent of the time for the occurrence of the four aquatic species groups and the five terrestrial species groups (Table 3). Since some empty cells may be empty because of insufficient collecting (false negatives), the correct prediction of actual occupancy (sensitivity in Table 3) is more informative about the performance of the models. Sensitivity ranges from 29.4 percent to 99.4 percent (Table 3). All but five of the 19 sensitivity measures were greater than 70 percent. The model did a poor job of predicting the presence/absence of pseudoscorpions in both regions and the presence/absence of endemics in both regions. Model fit, as measured by sensitivity, did not differ in the Interior Low Plateau and the Appalachian Mountains, and did not differ between aquatic and terrestrial species groups, and excluding pseudoscorpions and endemics, the overall mean was 87.6 percent (Table 3).

**Fig 3:**
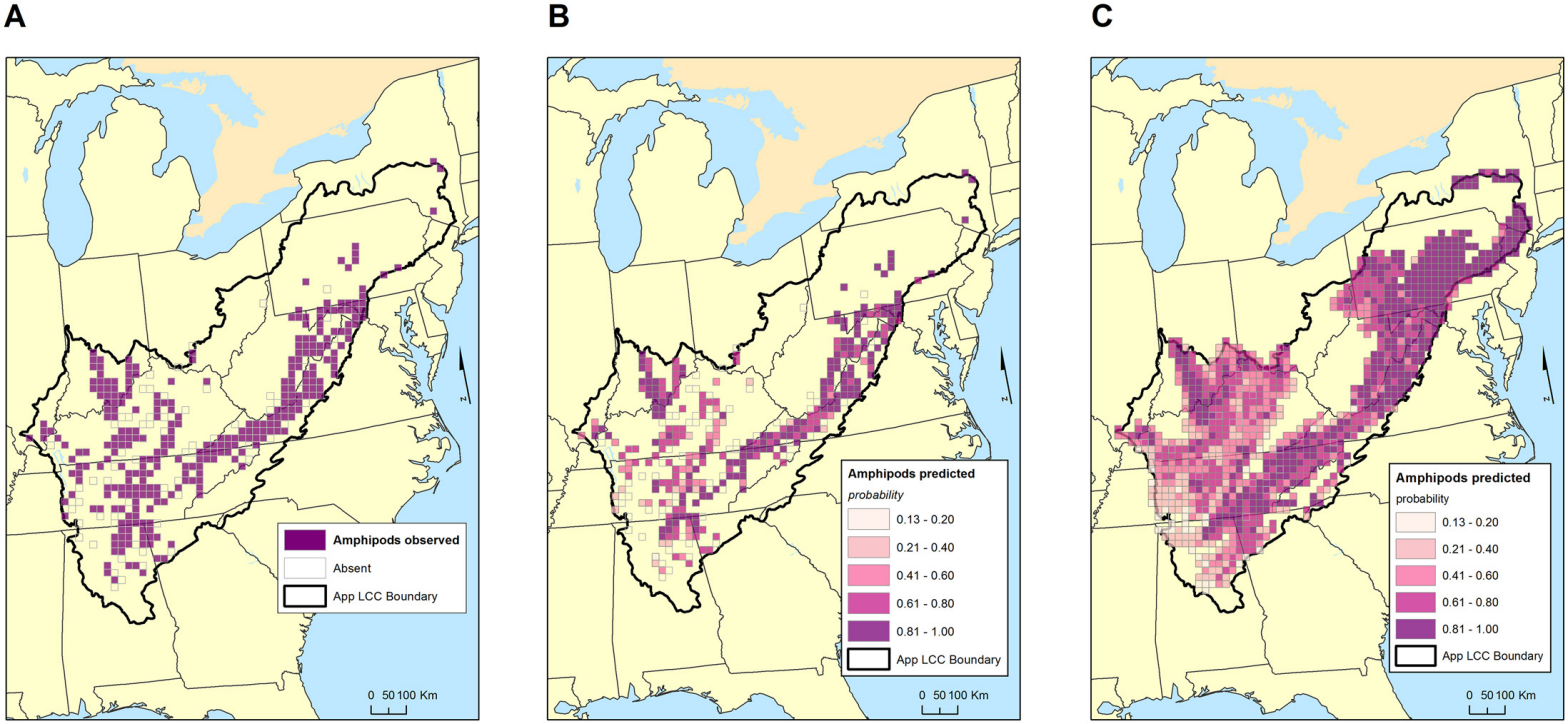
Maps of observed and predicted distribution of troglobiotic amphipods (largely the genera *Stygobromus* and *Crangonyx*) in the study area. A. Observed distribution of troglobiotic amphipods in 20 x 20 km grid. B. Predicted probabilities of occurrence of troglobiotic amphipods in those grid cells that have observed troglobionts. C. Predicted probabilities of occurrence of troglobiotic amphipods in all grid cells with karst. See Table 2 for details of the model and Table 3 for goodness of fit.

For amphipods in both the Appalachian Mountains and the Interior Low Plateau, amount of habitat (measured by percent karst), and both the mean and standard deviation of TPI (topographic position index) were associated with their presence. The probability of finding an amphipod in the Interior Low Plateau, but not the Appalachian Mountains, increases with latitude. The standard deviation of BFI (base flow index) is important in the Interior Low Plateau, and more diverse groundwater contributions to streams, which could reflect sensitivity to floods and droughts, decreases the likelihood of finding a troglobiotic amphipod. Temperature, precipitation, and elevation were important predictors of Appalachian Mountain amphipods. The correspondence between grid cells with troglobiotic amphipods (Fig 3A) and those predicted to have troglobiotic amphipods with a probability greater than 0.6 (Fig 3B) is high. For unsampled karst grid cells, Pennsylvania and New York are predicted to have amphipods in many grid cells (Fig 3C).

Isopods share many subterranean habitats with amphipods (especially streams and epikarst), and factors affecting the presence/absence of asellid isopods overlap with those predicting the presence/absence of amphipods. Shared predictors include mean temperature, precipitation, elevation, latitude, longitude, available habitat, and TPI. On the other hand, soil characteristic are important predictors for isopods but not amphipods (Table 2). Finally, above ground forest biomass was an important predictor of presence of troglobiotic isopods in the Interior Low Plateau. The distribution of observed asellid isopod occurrence and predicted probabilities of asellid isopod occurrence are shown in S1 Fig.

Only one crayfish species—*Cambarus nerterius*—is known from the Appalachian Mountains (southern West Virginia) so little inference can be made from the Appalachian regional analysis. Not surprisingly, geographic position is an important predictor. For the Interior Low Plateau, there are nine variables that add to the predictive power of the model. These include latitude, longitude, TPI, BFI, elevation, soil, and amount of available habitat (Table 2). The distribution of observed crayfish occurrence and predicted probabilities of crayfish occurrence are shown in S2 Fig.

Cavefish only occur in three grid cells in the Appalachian Mountains, so only the Interior Low Plateau was analyzed. Temperature, elevation, and precipitation were all important and somewhat surprisingly, probability of finding a cavefish declined with increasing temperature and showed a concomitant increase with latitude. Unlike the other aquatic groups, the length of the karst/non-karst edge had a negative effect (Table 2). The distribution of observed cavefish occurrence and predicted probabilities of cavefish occurrence are shown in S3 Fig.

**Fig 4:**
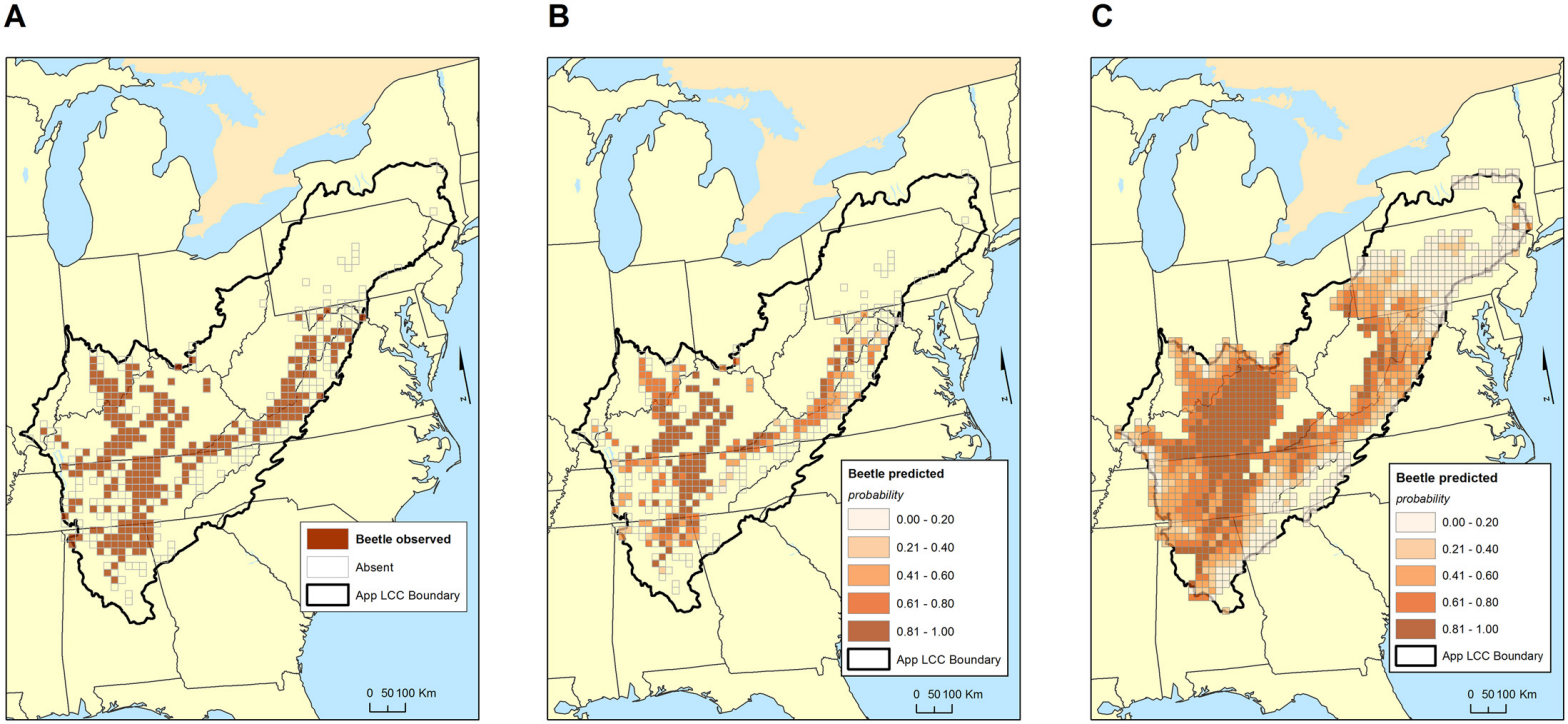
Maps of observed and predicted distribution of troglobiotic ground beetles (largely the genus *Pseudanophthalmus*) in the study area. A. Observed distribution of troglobiotic ground beetles in 20 x 20 km grid. B. Predicted probabilities of occurrence of troglobiotic ground beetles in those grid cells that have been observed beetles. C. Predicted probabilities of occurrence of troglobiotic ground beetles in all grid cells with karst. See Table 2 for details of the model and Table 3 for goodness of fit.

In both regions, mean elevation, and PC3 of soil (see Table 1) were important predictors of presence/absence of carabid beetles. Latitude was an important predictor for the Interior Low Plateau, but not for the Appalachian Mountains (Table 2). The correspondence between grid cells with troglobiotic ground beetles (Fig 4A) and those predicted to have troglobiotic amphipods with a probability greater than 0.6 (Fig 4B) is high. For unsampled karst grid cells, a number of grid cells in the Interior Low Plateau are expected to have ground beetles. It is interesting to note that most sampled grid cells in the Interior Low Plateau have ground beetles (Fig 2).

For both regions, above ground forest biomass (Mg/ha), latitude, and amount of habitat (percent karst) are important predictors of the presence/absence of millipedes (Table 2). In the case of latitude, the likelihood of finding millipedes increases, rather than decreases with increasing latitude. It is also noteworthy that variation in parameters (above ground biomass, TPI, precipitation, elevation, and BFI) has an impact on the probably of finding millipedes. In the case of TPI and elevation, the effect is positive; for the others it is negative. The distribution of observed millipede occurrence and predicted probabilities of millipede occurrence are shown in S4 Fig.

**Fig 5:**
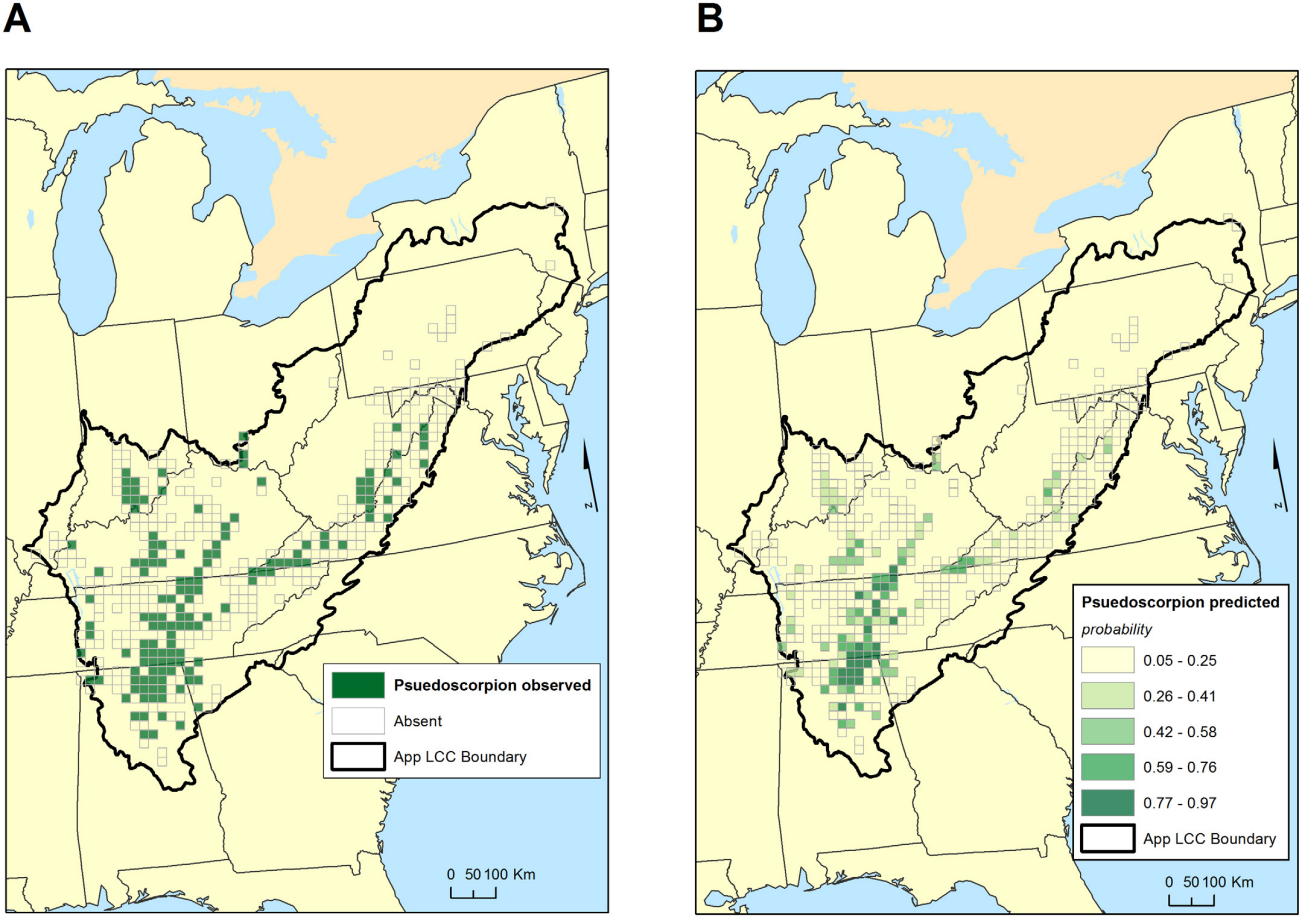
Maps of observed and predicted distribution of troglobiotic pseudoscorpions in the study area. A. Observed distribution of troglobiotic pseudoscorpions in 20 x 20 km grid. B. Predicted probabilities of occurrence of troglobiotic pseudoscorpions in those grid cells that have been sampled. Because of poorness of fit (Table 3), predicted probabilities of occurrence of troglobiotic pseudoscorpions in all grid cells with karst are not shown. See Table 2 for details of the model.

The models of pseudoscorpion occurrence for the two regions have little in common, sharing only a positive effect of the log of the length of the karst/non-karst boundary. The most significant aspect of the pseudoscorpion models is that they failed, in the sense that their ability to predict where pseudoscorpions were known from was very weak—only 34 to 40 percent of grid cells known to have troglobiotic pseudoscorpions were predicted to have them (Table 3). The distribution of observed pseudoscorpion occurrence and predicted probabilities of pseudoscorpion occurrence are shown in Fig 5. Since its predictive power is weak, predictions for unsampled karst grid cells are not shown.

Although both models have strong predictive power for spiders, there are no significant variables shared between the two regions (Table 2). The Appalachian Mountains are dominated by measures of topographic relief as significant variables, including elevation and TPI (both means and standard deviation); significant variables in the Interior Low Plateau are dominated by measures of the water budget—precipitation and BFI. The distribution of observed spider occurrence and predicted probabilities of spider occurrence are shown in S5 Fig.

In the case of Collembola, the two regions share two predictor variables—PC2 for soil and the standard deviation for TPI. For the Appalachian Mountains, elevation and TPI (both means and standard deviations) are important, while in the Interior Low Plateau, both latitude and karst/non-karst edge length are important, and the likelihood of finding springtails increases with latitude and decreases with increasing karst/non-karst edge length. The distribution of observed springtail occurrence and predicted probabilities of occurrence are shown in S6 Fig.

As was the case with pseudoscorpions, the model for endemics was essentially a failed model, only correctly predicting occurrences between 29 and 31 percent of the time (Table 3). Interestingly, latitude was not a significant factor in either the Appalachian Mountains or the Interior Low Plateau. The distribution of observed endemic occurrence and predicted probabilities of endemic occurrence are shown in S7 Fig.

## Discussion

### Biological Significance of Predictor Variables

Overall, we were successful in predicting the presence/absence of terrestrial and aquatic troglobiotic taxonomic groups in a 20 x 20 km grid overlying the karst regions of the Interior Low Plateau and Appalachian Mountains from predictors derived from the surface environment (Tables 2 and 3). This result re-emphasizes the strong dependence of cave fauna on surface features. These surface-subsurface connections take a variety of forms. Except in cases of chemoautotrophy [39], all organic matter in caves originates at the surface, and so cave fauna should be sensitive to changes in surface productivity (and the consequent subsurface inputs of organic matter, reflected in PC2 of the soil data). Temperature and precipitation set the broad outlines of the amount of surface primary productivity, and the moderate dependence on precipitation (both mean and standard deviation) and temperature (mean but rarely standard deviation) suggest the importance of productivity in determining the presence/absence of different groups of cave organisms (Table 2), a point also made by Culver et al. [16] in the context of terrestrial troglobiotic species richness in Europe and eastern U.S. Seasonality of both temperature and precipitation (expressed in their standard deviations) likewise influences productivity.

Elevation, together with latitude and longitude, is a controlling factor of temperature and precipitation. Mean elevation, but not its standard deviation, was often included in the list of significant predictor variables of species groups (Table 2); this may be another reflection of the importance of productivity. Another variable that may be connected with amount of organic carbon and nutrients available to cave-dwelling species is the standing crop biomass. Mean forest biomass (Mg/ha) was a moderately important predictor of presence/absence. Alternatively, current forests may be a good predictor of forests existing at the time troglobionts colonized karst in the region, and hence colonization potential. Forest litter is the habitat of many of the immediate ancestors of terrestrial cave species [9].

The soil is an important zone of processing of organic matter that enters caves through percolating water [40], and caves, especially those that are shallow, are embedded in a soil matrix, just as Gers [41] pointed out was the case for small cavities in talus slopes and elsewhere (the milieu souterrain superficiel). Which aspects of the soil are most important for the cave fauna remains elusive. The results presented here suggest that poorly drained soils with high organic content are unlikely to have terrestrial troglobionts (high loadings on PC2 [Table 1]). The connections between the soil and caves, both in terms of fauna and for energy transfer remain a largely unstudied area.

Other predictor variables, especially percent karst in a grid cell, are a measure of the amount of potential habitat. It was one of the most important predictor variables in this study (Table 2), and amount of habitat, measured either by percent karst or number of caves, has been found to be an important predictor variable of species richness in other regions and scales [30, 38, 42]. The variable designed to measure the degree of karst dissection—the length of the karst/non-karst contacts—proved to be generally unimportant (Table 3). On the other hand, one of the major differences between karst in the Appalachian Mountains and Interior Low Plateau is its degree of karst dissection—the Appalachian mountain karst is much more discontinuous [32]. It is possible that range sizes differ according to the degree of karst dissection, either at the local or regional scale, but that was not the focus of this study.

Both the mean base flow index (BFI) and its standard deviation were of moderate importance in predicting the presence of both terrestrial and aquatic groups. While BFI itself may be associated with productivity because of its connection with precipitation, the standard deviation of BFI is associated with variability in hydrogeologic conditions, reflecting sensitivity to floods and droughts. Flooding has a dual role in caves—it is an important source of organic matter, but it is also a cause of environmental stress [43]. In this study, the BFI had a negative effect on the likelihood of finding several species groups present (Table 3).

Latitude was often a predictor variable for the presence/absence of a species group, but it was not always the case that the probability decreased with increasing latitude. Species richness and perhaps numerical abundance of troglobionts typically reaches a maximum, not in the tropics, but in mid-temperate latitudes (e.g., the Pyrenees and Dinaric karst in Europe [16, 44]. However, we found that several groups, e.g., asellid isopods in both ecological regions, were more frequent in the northern part of the study area, e.g., Indiana and West Virginia). This may be the result of historical distribution patterns of surface progenitors or the effects of climate change during the Pleistocene, which are widely held to have forced species into caves [9, 45, 46] and which may have had an especially strong effect near the Pleistocene ice margin, as Barr [47] suggested for the Mitchell Plain in southern Indiana. There were also a few cases where longitude was a predictor of the presence/absence of groups (Table 3), with probability of presence decreasing in a westward direction (Table 2). Perhaps this is the result of a general decrease in precipitation with increase in longitude.

A final pair of variables, the mean and standard deviation of TPI (topographic position index) were predictors of presence/absence, especially the standard deviation of TPI which ranked first, along with percent karst as the most frequent predictor (Table 2). TPI is a measure of localized relief and so its standard deviation measures variability of relief, or rugosity. It is possible that rugosity in the landscape promotes cave development, but that remains to be demonstrated.

### Failures in Prediction—Pseudoscorpions and Endemics

We were unable to generate a predictive model for the presence/absence of pseudoscorpions or for endemic species (Table 3). Endemics and pseudoscorpions share an important feature—their frequency of grid occupancy is the lowest, except for fishes and crayfishes in the Appalachians (Fig 2). In the cases of fishes and crayfishes, there are likely historical reasons for their near absence in the Appalachians, while in the case of pseudoscorpions and endemics, it is likely to be restricted dispersal. Many pseudoscorpions are single grid cell or even single cave endemics. Christman et al. [11] report that 69 percent of pseudoscorpions from the eastern U.S. are single cave endemics, the highest reported taxon for any group except the poorly studied mites. Thus, it may be the case that pseudoscorpions are missing from many grid cells due to lack of colonization and/or dispersal. It may also be because pseudoscorpions are often in found in dry rather than wet organic matter. As a result, variation in pseudoscorpion species’ responses to environmental parameters makes model development difficult.

Endemics are by nature a different grouping, and by definition restricted in range. Somewhat paradoxically, the failure of the model to predict the occurrence of endemism may be because spatial effects (i.e., spatial autocorrelation), were not taken into account. Christman et al. [11] showed this to be the case in their study of terrestrial single cave endemics in the eastern U.S. The implication of their study is that endemism results from subterranean dispersal and isolation of peripheral populations, rather than direct colonization from the surface.

## Conclusions

The demonstration that surface features can be good predictors of the presence or absence of different groups of obligate cave-dwelling species is the most important finding of our study. The somewhat coarse scale of the analysis (20 x 20 km) was necessitated by the amount of data, but even at this relatively coarse scale, the model was predictive. It is perhaps disappointing that the connections were not simpler and more easily explainable, but this is not really surprising given the finding of multi-causality of Eme et al. [44] for groundwater crustacean biodiversity in Europe. The particular variables involved, especially TPI, suggest new avenues of approach to the study of the biogeography of the cave fauna. And finally, the models themselves provide a useful way for land managers to predict features of the subterranean fauna, before the laborious and expensive prospect of sampling. Such models should make identification and protection of the cave fauna both feasible and efficient.

## Supporting Information

S1 Fig Maps of observed and predicted distribution of troglobiotic isopods (largely the genus *Caecidotea*) in the study area. A. Observed distribution of troglobiotic isopods in 20 x 20 km grid. B. Predicted probabilities of occurrence of troglobiotic amphipods in those grid cells that have observed troglobionts. C. Predicted probabilities of occurrence of troglobiotic isopods in all grid cells with karst. See Table 2 for details of the model and Table 3 for goodness of fit.
(DOCX)

S2 Fig Maps of observed and predicted distribution of troglobiotic crayfish (largely the genera *Cambarus* and *Orconectes*) in the study area. A. Observed distribution of troglobiotic crayfish in 20 x 20 km grid. B. Predicted probabilities of occurrence of troglobiotic crayfish in those grid cells that have observed troglobionts. C. Predicted probabilities of occurrence of troglobiotic crayfish in all grid cells with karst. See Table 2 for details of the model and Table 3 for goodness of fit.
(DOCX)

S3 Fig Maps of observed and predicted distribution of troglobiotic fish (genera *Amblyopsis*, *Speoplatyrhinus* and *Typhlichthys*) in the study area. A. Observed distribution of troglobiotic fish in 20 x 20 km grid. B. Predicted probabilities of occurrence of troglobiotic fish in those grid cells that have observed troglobionts. C. Predicted probabilities of occurrence of troglobiotic fish in all grid cells with karst. See Table 2 for details of the model and Table 3 for goodness of fit. Because of the very small number of occurrences of fish in the Appalachian Mountains, they are not included in Tables 2 and 3.
(DOCX)

S4 Fig Maps of observed and predicted distribution of troglobiotic millipedes (genera *Pseudotremia*, *Scoterpes*, *Zygonopus*, and others) in the study area. A. Observed distribution of troglobiotic millipedes in 20 x 20 km grid. B. Predicted probabilities of occurrence of troglobiotic millipedes in those grid cells that have observed troglobionts. C. Predicted probabilities of occurrence of troglobiotic millipedes in all grid cells with karst. See Table 2 for details of the model and Table 3 for goodness of fit.
(DOCX)

S5 Fig Maps of observed and predicted distribution of troglobiotic spiders (including *Bathyphantes*, *Liocrinoides*, *Nesticus*, *Phanetta*, and *Porrhomma*) in the study area. A. Observed distribution of troglobiotic spiders in 20 x 20 km grid. B. Predicted probabilities of occurrence of troglobiotic spiders in those grid cells that have observed troglobionts. C. Predicted probabilities of occurrence of troglobiotic spiders in all grid cells with karst. See Table 2 for details of the model and Table 3 for goodness of fit.
(DOCX)

S6 Fig Maps of observed and predicted distribution of troglobiotic springtails (including *Pseudosinella*, *Pygmarrhopalites*, and *Sinella*) in the study area. A. Observed distribution of troglobiotic millipedes in 20 x 20 km grid. B. Predicted probabilities of occurrence of troglobiotic millipedes in those grid cells that have observed troglobionts. C. Predicted probabilities of occurrence of troglobiotic millipedes in all grid cells with karst. See Table 2 for details of the model and Table 3 for goodness of fit.
(DOCX)

S7 Fig Maps of observed and predicted distribution of single grid endemics in the study area. A. Observed distribution of troglobiotic endemics in 20 x 20 km grid. B. Predicted probabilities of occurrence of troglobiotic endemics in those grid cells that have observed troglobionts. C. Predicted probabilities of occurrence of troglobiotic endemics in all grid cells with karst. See Table 2 for details of the model and Table 3 for goodness of fit.
(DOCX)

## References

[1] Guisan A, Thuiller W. Predicting species distribution: offering more than simple habitat models. Ecol Lett. 2005;8: 993–1009. 10.1111/j.1461-0248.2005.00792.x 34517687

[2] Elith J, Leathwick JR. Species distribution models: ecological explanation and prediction across space and time. Ann Rev Ecol Evol Syst. 2009;40: 677–697.

[3] Guillera-Arroita G, Lahoz-Monfort JJ, Elith J, Gordon A, Kujala H, Lentini PE, et al Is my species distribution model fit for purpose? Matching data and models to applications. Global Ecol Biogeogr. 2015;24: 276–292.

[4] Sinclair SJ, White MD, Newell GR. 2010. How useful are species distribution models for managing biodiversity under future climates? Ecol Society, 2010;15: 8.

[5] Platts PJ, Garcia RA, Hof C, Foden W, Hansen LA, Rahbek C, et al Conservation implications of omitting narrow-ranging taxa from species distribution models, now and in the future. Diversity Distrib. 2014;20: 1307–1320.

[6] Ferrier S, Guisan A. 2006. Spatial modelling of biodiversity at the community level. J Appl Ecol. 2006;43: 393–404.

[7] Loarie SR, Carter BE, Hayhoe K, McMahon S, Moe R, Knight CA, et al Climate change and the future of California’s endemic flora. PLoS One 2008;3: e2502 10.1371/journal.pone.0002502 18648541 PMC2481286

[8] Ovaskainen O, Soininen J. Making more out of sparse data: hierarchical modeling of species communities. Ecology. 2011;92: 289–295. 21618908 10.1890/10-1251.1

[9] Peck SB. Climatic change and the evolution of cave invertebrates in the Grand Canyon. Bull Nat Speleological Soc. 1980;42: 53–60.

[10] Gibert J, Deharveng L. Subterranean ecosystems: a truncated functional biodiversity. Bioscience. 2002;52: 473–481.

[11] Christman MC, Culver DC, Madden M, White D. Patterns of endemism of the eastern North American cave fauna. J. Biogeogr. 2005;32: 1441–1452.

[12] Niemiller ML, Zigler KS. Patterns of cave biodiversity and endemism in the Appalachians and Interior Low Plateau of Tennessee, USA. PLoS One. 2013;8 10.1371/journal.pone.0064177 PMC3661478 23717562

[13] Malard F, Boutin C, Camacho AI, Ferreira D, Michel G, Sket B, et al Diversity patterns of stygobiotic crustaceans across multiple spatial scales in Europe.–Freshw. Biol. 2009;54: 756–776.

[14] Zagmajster M. Analiza razširjenosti izbranih skupin troglobiotske favne na Dinarskem območju. Ph.D. Dissertation, Univerza v Ljubljani, Slovenia. 2007.

[15] Faille A, Andujar C, Fadrique F, Ribera I. Late Miocene origin of an Iberio-Maghrebian clade of ground beetles with multiple colonizations of the subterranean environment. J Biogeogr. 2014;41: 1979–1990.

[16] Culver DC, Deharveng L, Bedos A, Lewis JJ, Madden M, Reddell JR, et al The mid-latitude biodiversity ridge in terrestrial cave fauna. Ecography. 2006;29: 120–128.

[17] Zagmajster M, Eme D, Fišer C, Galassi D, Marmonier P, Stoch F, et al Geographic variation in range size and beta diversity of groundwater crustaceans: insights from habitats with low thermal seasonality. Global Ecol Biogeogr. 2014;23: 1135–1145.

[18] Shunk AJ, Driese SG, Farlow JO, Zavada MS, Zobaa MK. Late Neogene paleoclimate and paleoenvironment reconstructions from the Pipe Creek Sinkhole, Indiana, USA: Palaeogeog Palaeoclim Palaeoecol 2009;274:,173–184,.

[19] Farlow JO, Steinmetz JC, DeChurch DA (eds.). Geology of the Late Neogene Pipe Creek Sinkhole (Grant County, Indiana), Indiana Geological Survey Special Report 2010; 69: 1–93

[20] Zobaa MK, Zavada MS, Whitelaw MJ, Shunk AJ, Oboh-ikuenobe FE. 2011, Palynology and palynofacies analyses of the Gray Fossil Site, eastern Tennessee: Their role in understanding the basin-fill history: Palaeogeog Palaeoclim Palaeoecol. 2011;308: 433–444, 10.1016/j.palaeo.2011.05.051

[21] Tschudy RH. An Upper Cretaceous deposit in the Appalachian Mountains. US Geol Survey Prof Paper. 1965;525-B: B64–B68.

[22] Niemiller ML, Fitzpatrick BM, Miller BT. Recent divergence with gene flow in Tennessee cave salamanders (Plethodontidae: *Gyrinophilus*) inferred from gene genealogies. Mol Evol. 2008:17: 2258–2275. 10.1111/j.1365-294X.2008.03750.x 18410292

[23] Niemiller ML, Miller BT, Fitzpatrick BM. Systematics and evolutionary history of subterranean *Gyrinophilus* salamanders. Proc 15^th^ Int Congr Speleol. 2009:15: 242–248.

[24] Niemiller ML, Fitzpatrick BM, Shah P, Schmitz L, Near TJ. Evidence for repeated loss of selective constraint in rhodopsin of amblyopsid cavefishes. Evolution. 2013:67: 732–748, 10.1111/j.1558-5646.2012.01822.x 23461324

[25] Niemiller ML, McCandless JR, Reynolds RG, Caddle J, Near TJ, Tillquist CR, et al Effects of climatic and geological processes during the Pleistocene on the evolutionary history of the northern cavefish, *Amblyopsis spelaea* (Teleostei: Amblyopsidae). Evolution. 2013:67: 1011–1025. 10.1111/evo.12017 23550752

[26] Corrigan LJ, Horton T, Fotherby H, White TA, Hoelzel AR. Adaptive evolution of deep-sea amphipods from the superfamily Lysiassanoidea in the North Atlantic. Evol Biol. 2014:41: 154–165.

[27] Taylor SJ, Niemiller ML. Biogeography and conservation assessment of Bactrurus groundwater amphipods (Crangonyctidae) in the central and eastern United States. Sub Biol. 2016:17: 1–29.

[28] Morvan C, Malard F, Paradis E, Lefébure T, Konecny-Dupré L, Douady CJ. Timetree of Aselloidea reveals species diversification dynamics in groundwater. Syst Biol: 2013:62: 512–522. 10.1093/sysbio/syt015 23461879

[29] Culver DC, Christman MC, Sket B, Trontelj P. Sampling adequacy in an extreme environment: species richness patterns in Slovenian caves. Biodiv Conserv. 2004;13: 1209–1229.

[30] Culver DC, Christman MC, Elliott WR, Hobbs HH III, Reddell JR. The North American obligate cave fauna: regional patterns. Biodiv Conserv. 2003;12: 441–468.

[31] Weary DJ, Doctor DH. 2014. Karst in the United States: A digital map compilation and database. USGS Open File Report 2014–1156. 2014; Available: http://pubs.usgs.gov/of/2014/1156/

[32] Barr TC. Observations on the ecology of caves. Am Nat. 1967;101: 475–492.

[33] Palmer A.N. and Palmer M.V. (eds.). Caves and karst of the USA. Huntsville: National Speleological Society; 2009.

[34] Ferrier S, Drielsma M, Manion G, Watson G. Extended statistical approaches to modelling spatial pattern in biodiversity in north-east New South Wales. II. Community-level modelling. Biodiv Conserv. 2002;11: 2309–2338.

[35] Jenness J, Brost B, Beier P. Land facet corridor designer: Extension for ArcGIS. Jenness Enterprises. 2013; Available: http://www.jennessent.com/arcgis/land_facets.htm

[36] Wolock DM. Flow characteristics at U.S. Geological Survey streamgages in the conterminous United States.—USGS Opean File Report 03–146. 2003; Available: http://water.usgs.gov/lookup/getspatial?qsitesdd.

[37] Hosmer DW Jr, Lemeshow S. Applied logistic regression, 2nd edition New York: John Wiley and Sons; 2000.

[38] Christman MC, Culver DC. The relationship between cave biodiversity and available habitat. J Biogeogr. 2001;28: 367–380.

[39] Engel AS. Chemoautotrophy In: White WB, Culver DC, editors. Encyclopedia of caves, second edition Amsterdam: Elsevier/Academic Press; 2012 pp. 125–134.

[40] Simon KS, Pipan T, Ohno T, Culver DC. Spatial and temporal patterns in abundance and character of dissolved organic matter in two karst aquifers. Fund Appl Limnol. 2010;177: 81–92.

[41] Gers C. Diversity of energy fluxes and interactions between arthropod communities, from soil to cave. Acta Oecologia. 1998;19: 205–213.

[42] Graening GO, Fenolio DB, Slay ME. 2011 Cave life of Oklahoma and Arkansas Exploration and conservation of subterranean biodiversity. Norman: University of Oklahoma Press; 2011.

[43] Hawes RW. The flood factor in the ecology of caves. J Anim Ecol. 1939;8: 1–5.

[44] Eme D, Zagmajster M, Fišer C, Galassi D, Marmonier P, Stoch F, et al Multi-causality and spatial non-stationarity in the determinants of groundwater crustacean diversity in Europe. Ecography. 2015;38: 511–540.

[45] Jeannel R. Les fossiles vivants des cavernes. Paris: Gallimard; 1943.

[46] Barr TC. Cave ecology and the evolution of troglobites. Evol Biol. 1968;2: 35–102.

[47] Barr TC. Synopsis of the cave beetles of the Genus Pseudanophthalmus of the Mitchell Plain in southern Indiana (Coleoptera, Carabidae). Am Midland Nat. 1960;63: 307–320.

[48] Doctor DH, Young JA, Christman M, Niemiller M, Zigler K, Weary DJ, Culver D. GIS data for predicting the occurrence of cave-inhabiting fauna based on features of the Earth surface environment in the Appalachian Landscape Conservation Cooperative (LCC) Region: U.S. Geological Survey: Reston, VA, 2016, 10.1371/journal.pone.0160408 PMC4988700 27532611

