# Supplementary material for: Predicting the Occurrence of Cave-Inhabiting Fauna Based on Features of the Earth Surface Environment
Source: PLoS One. 2016 Aug 17;11(8):e0160408. doi: 10.1371/journal.pone.0160408 (PMC4988700; doi:10.1371/journal.pone.0160408)

**S3 Fig. Maps of observed and predicted distribution of troglobiotic fish (genera *Speoplatyrhinus* and *Typhlichthys*) in the study area.** A. Observed distribution of troglobiotic fish in 20 X 20 km grid. B. Predicted probabilities of occurrence of troglobiotic fish in those grid cells that have observed troglobionts. C. Predicted probabilities of occurrence of troglobiotic fish in all grid cells with karst. See Table 2 for details of the model and Table 3 for goodness of fit. Because of the very small number of occurrences of fish in the Appalachian Mountains, they are not included in Tables 2 and 3.


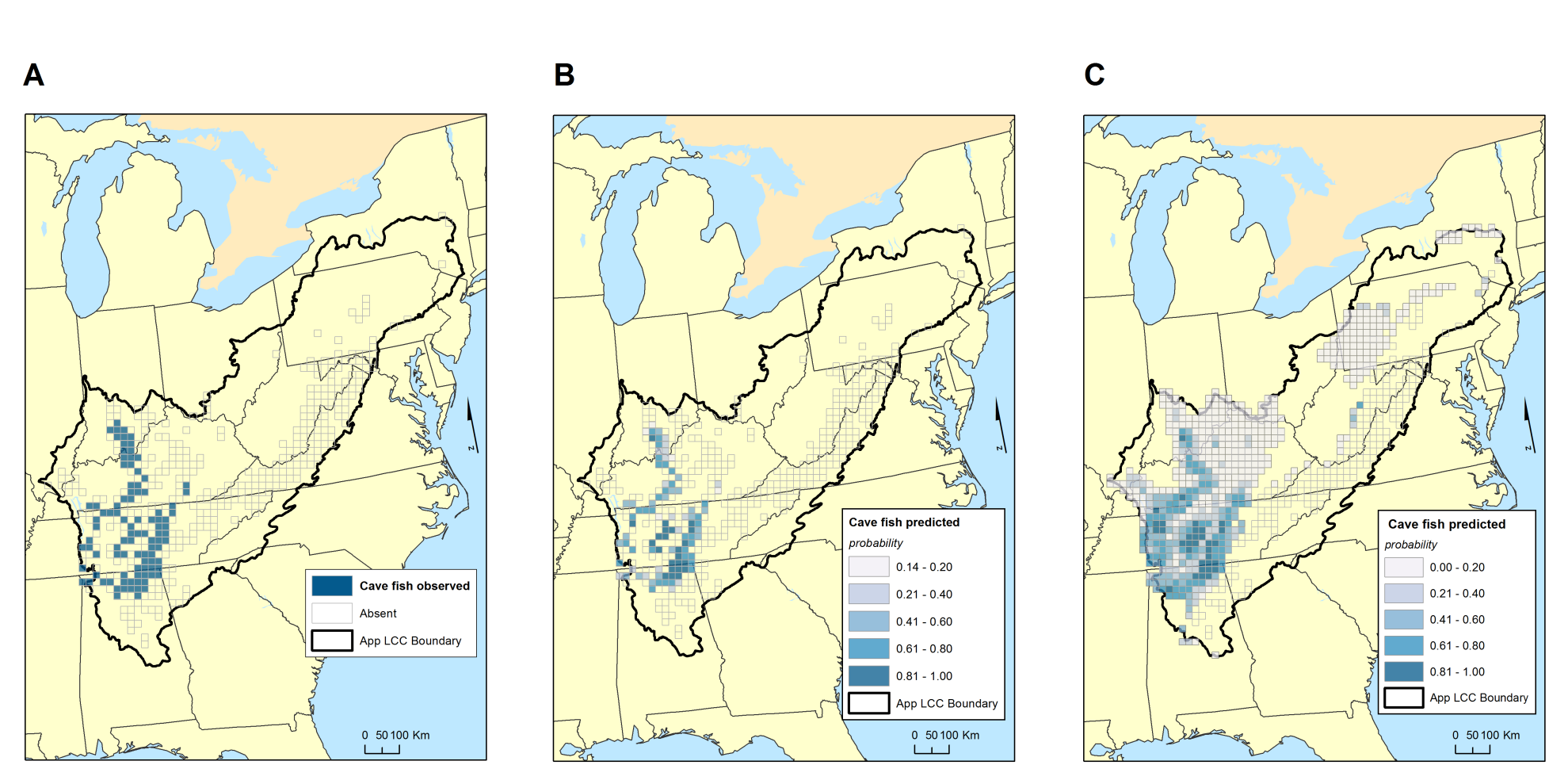

Supplement: S3 Fig — A. Observed distribution of troglobiotic fish in 20 x 20 km grid. B. Predicted probabilities of occurrence of troglobiotic fish in those grid cells that have observed troglobionts. C. Predicted probabilities of occurrence of troglobiotic fish in all grid cells with karst. See Table 2 for details of the model and Table 3 for goodness of fit. Because of the very small number of occurrences of fish in the Appalachian Mountains, they are not included in Tables 2 and 3. (DOCX) [file pone.0160408.s003.docx]
