# Supplementary material for: Predicting the Occurrence of Cave-Inhabiting Fauna Based on Features of the Earth Surface Environment
Source: PLoS One. 2016 Aug 17;11(8):e0160408. doi: 10.1371/journal.pone.0160408 (PMC4988700; doi:10.1371/journal.pone.0160408)

**S4 Fig. Maps of observed and predicted distribution of troglobiotic millipedes (genera *Pseudotremia, Scoterpes,* *Zygonopus*, and others) in the study area.** A. Observed distribution of troglobiotic millipedes in 20 X 20 km grid. B. Predicted probabilities of occurrence of troglobiotic millipedes in those grid cells that have observed troglobionts. C. Predicted probabilities of occurrence of troglobiotic millipedes in all grid cells with karst. See Table 2 for details of the model and Table 3 for goodness of fit.


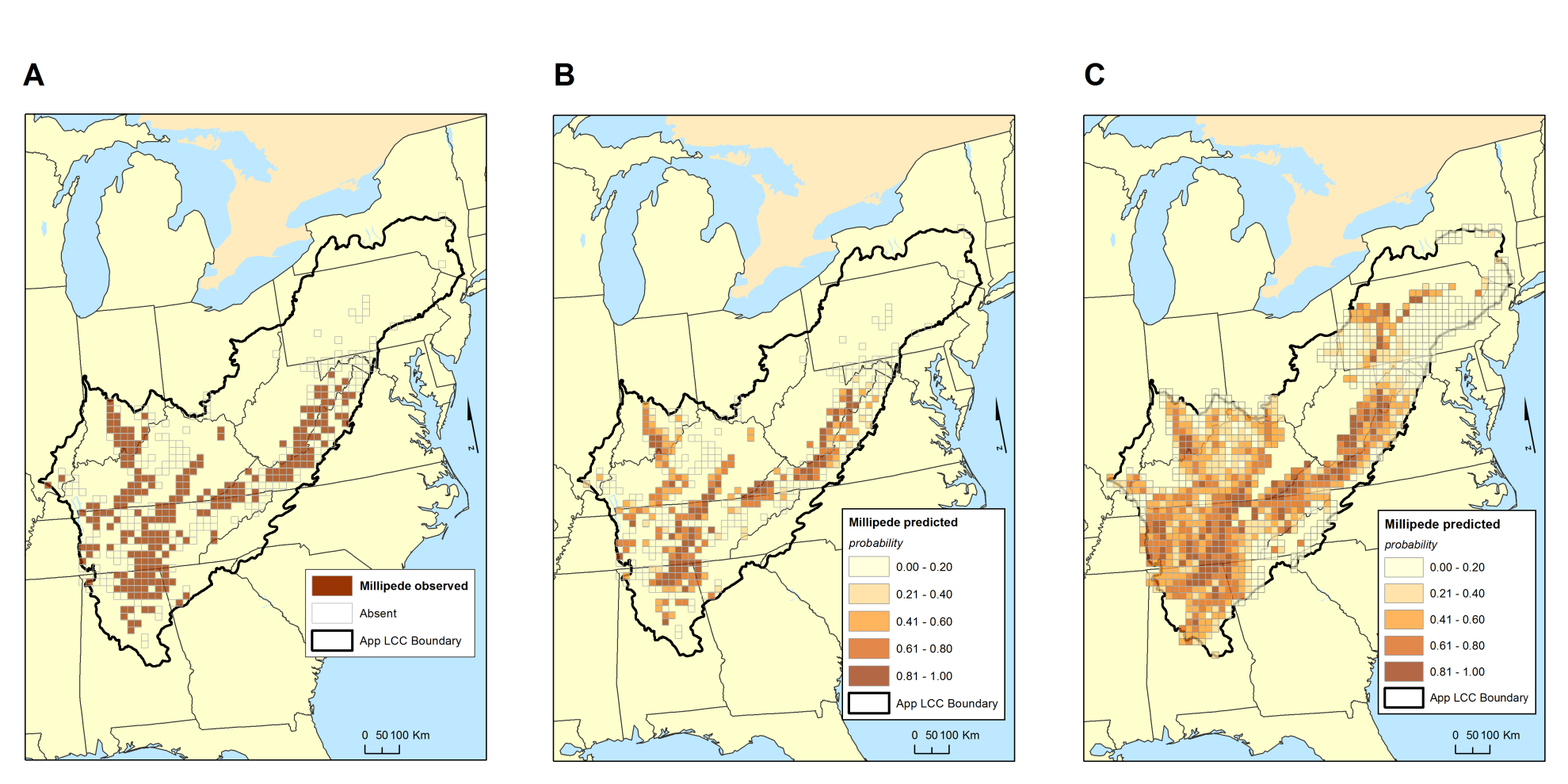

Supplement: S4 Fig — A. Observed distribution of troglobiotic millipedes in 20 x 20 km grid. B. Predicted probabilities of occurrence of troglobiotic millipedes in those grid cells that have observed troglobionts. C. Predicted probabilities of occurrence of troglobiotic millipedes in all grid cells with karst. See Table 2 for details of the model and Table 3 for goodness of fit. (DOCX) [file pone.0160408.s004.docx]
